# Supplementary material for: The β-lactam adjuvant guanosine potentiates anti-folate antibiotics and pyrimidine synthesis inhibitors by depleting thymidine in methicillin-resistant Staphylococcus aureus
Source: bioRxiv. 2026 Jan 15:2026.01.15.699642. Preprint. [Version 1] doi: 10.64898/2026.01.15.699642 (PMC12871101; doi:10.64898/2026.01.15.699642)
Supplement: 1 [file NIHPP2026.01.15.699642V1-supplement-1.pdf]

bioRxiv preprint doi: https://doi.org/10.64898/2026.01.15.699642; this version posted January 15, 2026. The copyright holder for this preprint (which was not certified by peer review) is the author/funder, who has granted bioRxiv a license to display the preprint in perpetuity. It is made available under aCC-BY 4.0 International license.

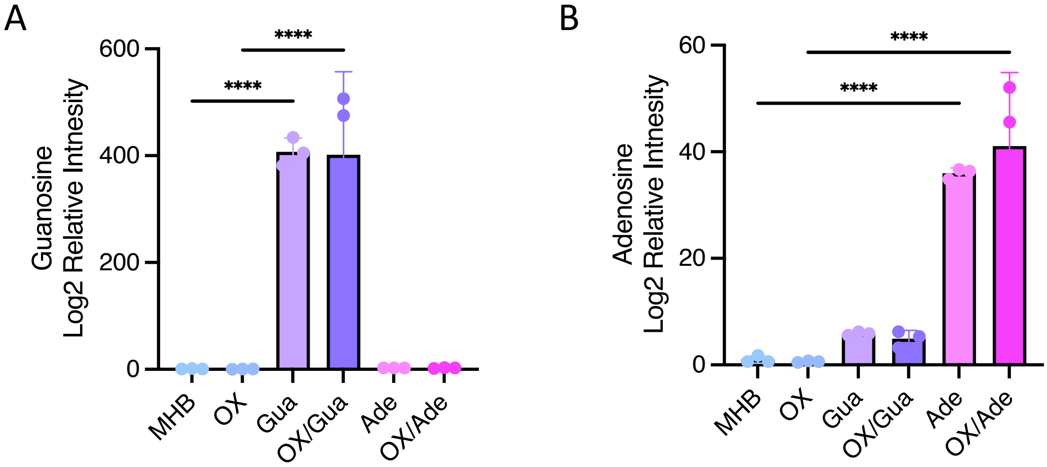

Fig. S1. Impact of exogenous guanosine or adenosine alone and in combination with oxacillin on intracellular guanosine and adenosine in MRSA strain JE2. A. Intracellular guanosine (A) and adenosine (B) were quantified in cells grown in MHB, MHB oxacillin (OX, 1 μg/ml), MHB guanosine (Gua, 200 μg/ml), MHB OX/Gua, MHB adenosine (Ade, 200 μg/ml) and MHB OX/Ade. Data presented are the average of three biological replicates plotted using GraphPad Prism V9. Asterisks indicate statistically significant difference according to using a one-way ANOVA test. p-values **** p<0.0001 are indicated.

bioRxiv preprint doi: https://doi.org/10.64898/2026.01.15.699642; this version posted January 15, 2026. The copyright holder for this preprint (which was not certified by peer review) is the author/funder, who has granted bioRxiv a license to display the preprint in perpetuity. It is made available under aCC-BY 4.0 International license.

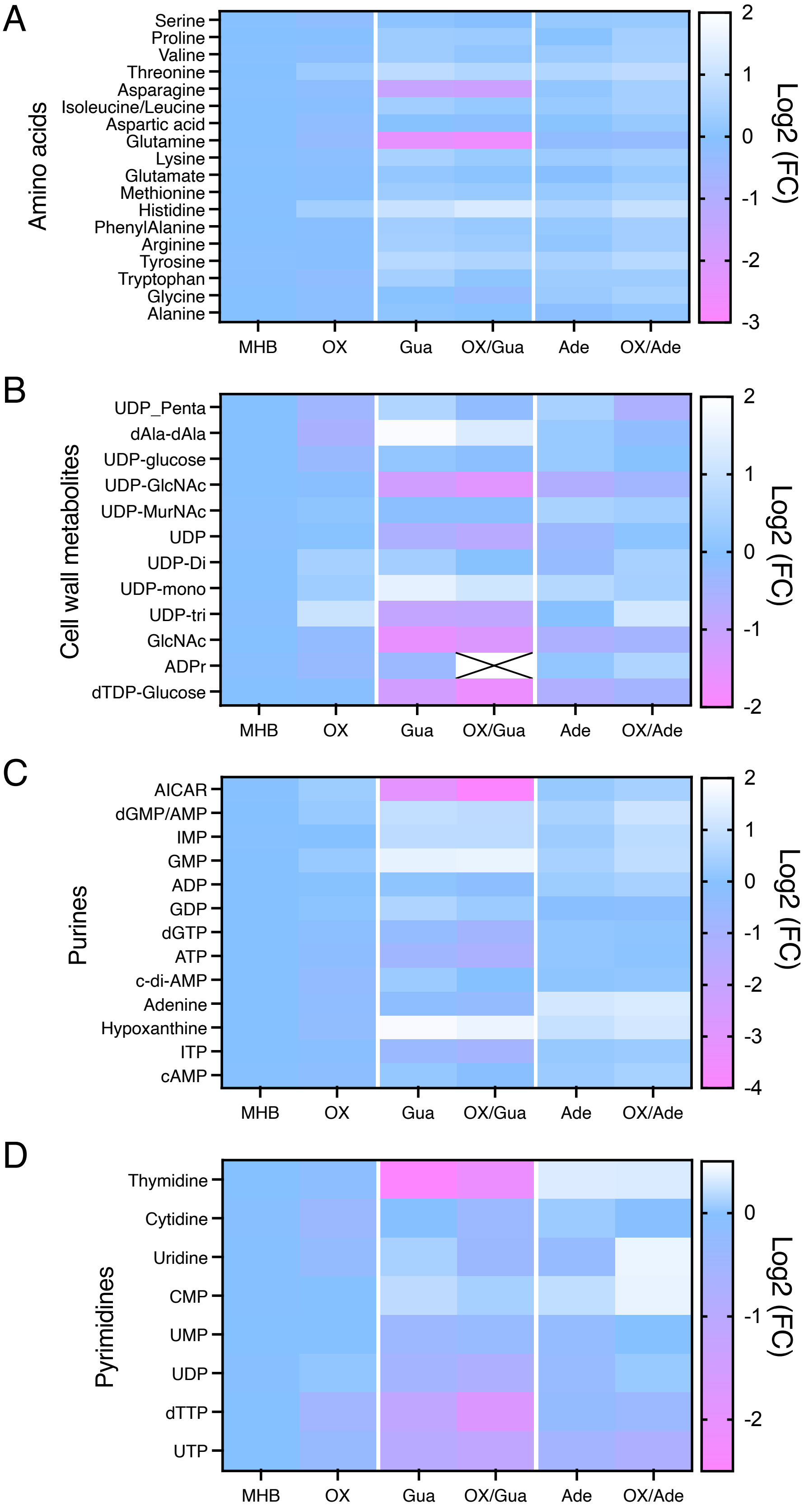

Fig. S2. Exposure of MRSA to guanosine or adenosine has pleiotropic effects on the MRSA metabolome. Heatmap comparison of amino acids (A), cell wall metabolites (B), purines (C) and pyrimidines (D) in JE2 grown in MHB and MHB supplemented with guanosine

bioRxiv preprint doi: https://doi.org/10.64898/2026.01.15.699642; this version posted January 15, 2026. The copyright holder for this preprint (which was not certified by peer review) is the author/funder, who has granted bioRxiv a license to display the preprint in perpetuity. It is made available under aCC-BY 4.0 International license.

Gua/antimetabolite combinations; **G.** SMX ± OX, 5-FU and Gua, **H.** SMX ± OX, 5-FUrd and Gua; **I.** TMP ± OX, 5-FU and Gua and **J.** TMP ± OX, 5-FUrd and Gua. Exponential phase cultures were inoculated into MHB 2% NaCl at a starting cell density of approximately 1×10^6 CFU/ml, with or without antibiotics and Gua as indicated, and CFUs enumerated after 0, 2, 4, 6, 8, 12, and 24 h. The data presented are the average of three independent experiments plotted using GraphPad Prism V9 and standard deviations are shown.

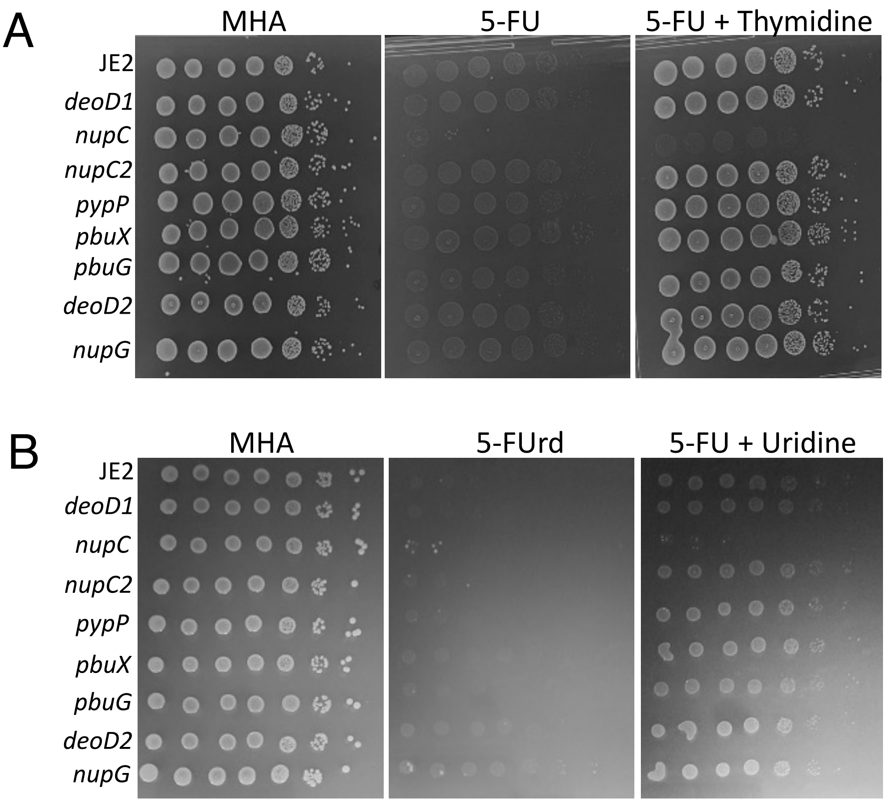

Fig. S3. Thymidine or uridine rescue MRSA growth inhibition by 5-fluorouracil (A) and 5-fluoruridine (B), respectively, in a *nupC*-dependent manner. Serial dilution spot plate assays using NTML mutants implicated in nucleotide transport and metabolism on Mueller-

bioRxiv preprint doi: https://doi.org/10.64898/2026.01.15.699642; this version posted January 15, 2026. The copyright holder for this preprint (which was not certified by peer review) is the author/funder, who has granted bioRxiv a license to display the preprint in perpetuity. It is made available under aCC-BY 4.0 International license.

Table S1. Antibacterial activity (zone diameters, mm) and synergy of cloxacillin (Clox), sulfamethoxazole (SMX), trimethoprim (TMP), 5-fluorouracil (5-FU), 5-fluorouridine (5-FUrd), and Bactrim (Bac), alone and in combination with guanosine (Gua, 200 μg/ml), against MRSA strains JE2, MW2, COL, and BH1CC.

Strain / Drug +/- Gua	JE2	MW2	COL	BH1CC
Clox	11	6	6	6
Clox/Gua	29	12	6	9
SMX	6	6	6	6
SMX/Gua	15	6	6	14
TMP	19	18	17	19
TMP/Gua	20	20	21	21
5-FU	14	6	11	19
5-FU/Gua	17	12	25	19
5-FUrd	15	11	16	16
5-FUrd/Gua	16	17	24	20
Double drug combinations +/- Gua[1]				
SMX-TMP	+	+	+	+
SMX-TMP/Gua	++	(+)	++	(+)
Clox-5-FU	+	-	+	-
Clox-5-FU/Gua	++	++	++	++
Clox-5-FUrd	+	+	-	-
Clox-5-FUrd/Gua	++	++	++	++
SMX-5-FU	+	-	-	-
SMX-FU/Gua	++	-	++	++
SMX-5-FUrd	+	-	-	-
SMX-5-FUrd/Gua	++	++	++	++
TMP-5-FU	+	-	+	+
TMP-5-FU/Gua	(+)	++	++	++
TMP-5-FUrd	+	+	+	+
TMP-5-FU/Gua	(+)	++	++	++

[1] Synergy was assessed by placing two antibiotic-impregnated disks 10 mm apart on MHA plates and defined as an increased zone of inhibition in the region between the disks relative to single-drug controls. "-" no detectable synergy; "+" synergy in the absence of Gua; "(+)" no increase in synergy in the presence of Gua; "++" enhanced synergy between the two drugs in the presence of Gua.

bioRxiv preprint doi: https://doi.org/10.64898/2026.01.15.699642; this version posted January 15, 2026. The copyright holder for this preprint (which was not certified by peer review) is the author/funder, who has granted bioRxiv a license to display the preprint in perpetuity. It is made available under aCC-BY 4.0 International license.

Table S2. Bacterial strains and plasmids used in this study

Strains	Relevant Details
JE2	USA300 cured of p01 & p03. Parent of Nebraska Transposon Mutant Library (NTML).
MW2	MRSA SCCmec type IV; CC1 (65)
COL	MRSA reference strain; SCC*mec* type I; CC8 (66)
BH1CC	MRSA clinical isolate; SCC*mec* type II; CC8 (67)
NE1419 *nupG*	JE2 *nupG* (SAUSA300_0611). Erm^{r}. (68)
NE283 *pbuG/stgP*	JE2 *pbuG/stgP* (SAUSA300_2207). Erm^{r}. (68)
NE280 *pbuX*	JE2 *pbuX* (SAUSA300_0387). Erm^{r}. (68)
NE650 *deoD2*	JE2 *deoD2* (SAUSA300_2091). Erm^{r}. (68)
NE477 *deoD1*	JE2 *deoD1* (SAUSA300_0138). Erm^{r}. (68)
NE544 *nupC1*	JE2 *nupC1* (SAUSA300_0298). Erm^{r}. (68)
NE622 *nupC2*	JE2 *nupC2* (SAUSA300_0313). Erm^{r}. (68)
NE1048 *pyrP*	JE2 *pyrP* (SAUSA300_1092). Erm^{r}. (68)
